# Agreement and Reliability of Running Stride-Time Variability Analyses from Wearable Devices

**DOI:** 10.3390/s26113407

**Published:** 2026-05-28

**Authors:** Ben D. M. Jones, Jon Wheat, Kane Middleton, David L. Carey, Ben Heller

**Affiliations:** 1 Sport and Physical Activity Research Centre, Sheffield Hallam University, Olympic Legacy Park, 2 Old Hall Rd, Sheffield S9 3TY, UK; b.d.jones@shu.ac.uk; 2 Sport, Performance, and Nutrition Research Group, School of Allied Health, Human Services and Sport, La Trobe University, Melbourne, VIC 3086, Australia; k.middleton@latrobe.edu.au (K.M.); d.carey@latrobe.edu.au (D.L.C.); 3 Sport and Human Performance Enhancement Research Centre, Nottingham Trent University, Clifton Campus, Nottingham NG11 8NS, UK; jon.wheat@ntu.ac.uk

**Keywords:** gait variability, running, wearable devices, event detection, agreement, reliability

## Abstract

**Highlights:**

**What are the main findings?**

- Agreement of running stride-time variability measures derived from wearable devices with those derived from force plates ranged from moderate to excellent.
- Between-day reliability for each stride-time variability measure was fairly consistent across devices.

**What is the implication of the main finding?**

- Wearables can facilitate running stride-time variability analyses, with suitability depending on the device, and changes over time requiring cautious interpretation.

**Abstract:**

Stride-time variability is typically quantified using measures including the coefficient of variation (CV) and detrended fluctuation analysis (DFA). Whilst several studies have reported guidelines for applying such measures, few have considered the sensitivity of these measures to the way stride times are obtained. This study investigated the agreement and between-day reliability of stride-time variability measures derived from wearable devices compared to measures derived from an instrumented treadmill. Thirty-one runners completed eight minutes of running on two days. Stride times were obtained concurrently using Loadsol^®^ insoles, Blue Trident inertial measurement units (IMUs) at four sampling rates, RunScribe™ IMUs and an AMTI instrumented treadmill. Stride-time CV and DFA-α were calculated on time-matched series. Agreement with the instrumented treadmill was quantified using Bland Altman plots and concordance correlation coefficients. Loadsol^®^ insoles and RunScribe™ IMUs displayed the highest and lowest agreement, respectively. For the Blue Trident IMU, sampling below 400 Hz reduced agreement relative to higher rates. Between-day reliability was moderate-to-good and poor-to-moderate for stride-time CV and DFA-α, respectively, although within narrower bands than reported across studies using different measurement devices. Hence, Loadsol^®^ insoles and Blue Trident IMUs, at sufficient sampling rates, can facilitate stride-time variability analyses, although changes over time should be interpreted cautiously.

## 1. Introduction

Running-related injury (RRI) incidence is high [1], but evidence for a linear relationship between biomechanical variables and RRI remains limited [2,3], possibly because human movement is inherently variable. When attempting to achieve the same task goal, even elite performers display intra-individual variability [4]. This variability helps individuals adapt to dynamic individual, environmental and task constraints, and may be associated with human health [5,6]. Within running, variability refers to changes in an individual’s gait over successive gait cycles [7].

A notable feature of gait variability is variation in the duration of gait cycles, referred to as stride-time variability [8]. Although other biomechanical variables display variability during running [9,10], stride times are a convenient measure since they are easy to measure and require minimal data processing. Stride-time variability encompasses both the magnitude and structure of variability within stride-time series [11]. The magnitude of stride-time variability has typically been quantified using linear measures such as standard deviation (SD) and coefficient of variation (CV) [11]. Conversely, the structure of variability has been quantified using non-linear methods, such as detrended fluctuation analysis (DFA) [12]. DFA evaluates the temporal structure of fluctuations in time series and returns a scaling exponent, α, quantifying statistical persistence [13]. An α value < 0.5 indicates anti-persistence whereas α value between 0.5 and 1.0 indicates persistence. DFA has been used to identify disease and pathological alterations of gait [14], and may be more sensitive to changes in human health than linear measures [12]. For example, Meardon et al. [15] found stride-time DFA, but not SD nor CV, could distinguish between previously injured and uninjured groups during a 5-km run.

All stride-time variability measures require the accurate identification of a consistent event during the gait cycle. For example, stride times may be calculated as the time between consecutive foot contact events. Instrumented treadmills are typically used as the gold standard method for identifying foot contact events [16]. However, they are expensive and immobile, which creates challenges for participant recruitment and monitoring, and limits stride-time analyses to laboratory settings [17]. This raises concerns about the representativeness of analyses, since runners typically train in outdoor settings [18] and running biomechanics may differ between treadmill and overground locomotion [19].

Wearable devices offer a cheap and accessible option to conduct longitudinal studies monitoring stride-time variability in representative field-based settings [17]. Using inertial measurement units (IMUs), stride times can be calculated as the intervals between peak values in acceleration signals [15]. Using force-sensitive insoles, vertical force thresholds can be used to define touchdown events [20]. Moreover, some commercial wearable devices, such as RunScribe™ IMUs, provide pre-processed stride-time data, removing the need to handle raw data signals.

Before wearable devices can be used in field-based settings, an understanding of the sensitivity of stride-time variability measures to measurement method is required. However, despite reported guidelines on how to improve the consistency of stride-time variability measures such as DFA [21,22], little consideration has been given to the sensitivity of these measures to how stride-time data is obtained. Indeed, the agreement in mean stride time between measurement devices does not necessarily imply agreement in stride-time variability measures, particularly those that consider the temporal structure of time series [8]. Simulations have shown that adding noise-to-foot contact events reduces the statistical persistence of stride-time series whilst leaving the mean stride time unchanged [23]. Experimentally, motion-capture-based methods of stride-time detection have been shown to produce systematically higher estimates of DFA-α compared to an instrumented treadmill [23], despite demonstrating excellent agreement for stride-time mean and variance. It has thus been recommended that researchers ensure new technologies produce valid and reliable estimates of DFA-α relative to reference devices. The sampling rate of a device must also be considered when quantifying stride-time variability, since reduced sampling frequencies can result in underestimations of stride time DFA-α relative to higher frequencies [23,24]. Since lower sampling rates would be more practical for longitudinal data collection with wearable devices, it is also important to test the agreement and reliability of new technologies across a range of sampling frequencies to find a compromise between practicality and validity.

For wearable devices, the effect of stride-time detection method on variability measures during running remains unexplored. For walking gait, a systematic review by Kobsar et al. [25] reported poor-to-excellent validity in linear measures of step-time variability but found no evidence of research into the validity of non-linear measures. Recently, though, Slattery et al. [8] found walking stride-time variability measures from a range of wearable devices to display moderate-to-excellent agreement against an instrumented treadmill, with linear measures displaying the greatest agreement. For running gait, Zignoli et al. [26] reported differences in stride-time variability measures between an IMU and Optogait system over multiple treadmill speeds and gradients. Nevertheless, given the differences in measures of stride-time variability between walking and running [27], an investigation into the differences in stride times and stride-time variability measures between the instrumented treadmill and a range of wearable devices appears warranted.

Understanding the differences between non-linear variability measures, such as DFA-α, from different stride-time measurement devices may also yield insight into the reported differences in between-day reliability. Between-day reliability of running stride-time DFA-α has been reported as good when using an Optogait system [28] and force-sensitive resistors [29] but poor when using RunScribe™ IMUs [30]. When using a motion capture system, between-day reliability of stride-time DFA-α has ranged from poor to good as running duration increases [31], despite always meeting guidelines for minimum series length [32]. By comparison, between-day reliability of linear measures of stride-time variability is typically higher [29,30,31].

The aim of this study was thus two-fold. Firstly, to assess the agreement in running stride times and stride-time variability between an instrumented treadmill and a set of wearable devices (IMU generating raw acceleration data, force-sensing insoles, commercial IMU generating processed stride-time data) sampling at a range of frequencies (200–1600 Hz). Secondly, to assess the test-retest reliability of stride-time variability measures from different devices during treadmill running. We hypothesised that agreement with the instrumented treadmill would be greatest for the force-sensing insoles because both systems quantify ground reaction forces, that agreement would reduce with sampling rate, and that between-day reliability would be greater for linear measures of stride-time variability.

## 2. Materials and Methods

### 2.1. Participants

Thirty-one recreational adult runners (21 male, 10 female; mean ± SD: age 29.1 ± 7.5 years, mass 71.5 ± 11.7 kg, height 1.74 ± 0.09 m) provided written informed consent to participate in a protocol approved by the La Trobe University Human Research Ethics Committee (HEC24378) and Sheffield Hallam University Ethics Committee (ER72264498). Participants were required to be running a minimum of twice per week for at least 20 min per run, and to have experienced no running-related musculoskeletal injuries within the previous three months.

### 2.2. Instrumentation

Loadsol^®^ insoles (novel GmbH, Munich, Germany), sampling at 200 Hz were placed inside participants’ shoes. A Blue Trident IMU (Vicon Motion Systems Ltd., Oxford, UK) sampling acceleration data at 1600 Hz was secured to the anteromedial aspect of participants’ left tibia [15]. A RunScribe™ IMU (Scribe Labs, Moss Beach, CA, USA) sampling at 500 Hz and programmed by RunScribe™ (RunScribe™ Blue Plus, 37.53) with proprietary algorithms was placed in a lace cradle on the participant’s left and right running shoes. Devices were calibrated according to the manufacturer’s instructions.

### 2.3. Protocol

Participants completed an eight-minute warm-up at a self-selected running speed, which also served as a period of familiarisation to running on a tandem instrumented treadmill [19] (AMTI Inc., Watertown, MA, USA). Following a left-footed stamp—an event used for device synchronisation—and 15 s of gait initiation, participants completed eight minutes of treadmill running at 85% of a self-reported 5-km speed, which included at least 560 strides. Force data were collected by force plates integrated into the treadmill at 1000 Hz via Vicon Nexus software (version 2.16). Data were collected with the Loadsol^®^ insoles, Blue Trident IMUs and RunScribe™ IMUs using Loadapp (version 3.2.0), CaptureU (version 1.4.1) and RunScribe™ (version 2.4.7) applications respectively. Participants repeated the trial seven days later.

### 2.4. Data Processing

Data were downloaded from all applications and imported into MATLAB (R2023b, Mathworks Inc, Natick, MA, USA) for processing.

#### 2.4.1. Instrumented Treadmill and Loadsol^®^ Insoles

Vertical ground reaction force data from the treadmill and Loadsol^®^ insoles were filtered using a recursive second-order low-pass Butterworth filter at cutoff frequencies of 40 Hz and 20 Hz respectively following a residual analysis and visual inspection. A 50-N threshold was used to define initial contact. Stride times were calculated as the time between consecutive ipsilateral initial contact events.

#### 2.4.2. Blue Trident IMUs

High-*g* accelerometer data from the Blue Trident IMUs were downsampled to 800, 400 and 200 Hz by extracting every *i*th data point. These data were filtered using a recursive second-order low-pass Butterworth filter at a cutoff frequency of 50 Hz following a residual analysis and visual inspection. For each sampling rate, resultant acceleration was found as the Euclidean norm of *x*, *y* and *z* components. Stride times were defined as the time between consecutive peaks from initial contact events in resultant acceleration signals [33].

#### 2.4.3. RunScribe™ IMUs

Every trial of processed stride-time data downloaded from the RunScribe™ dashboard contained at least one skipped or erroneous stride. Overall, 3.5% of strides were skipped or erroneously reported. These were uniformly distributed throughout trials and evenly split between left and right feet, as previously reported [30]. Missing or erroneous stride-time data were removed and valid stride times were concatenated to form a single series since stride-time series were expected to display statistical persistence and thus to be robust to data loss [30,34].

### 2.5. Data Analysis

Data from the treadmill, Loadsol^®^ insoles and Blue Trident IMUs were time synchronised using the stamp event. As the RunScribe™ proprietary algorithms filtered out the stamp, stride times from the RunScribe™ IMUs were time synchronised with the instrumented treadmill stride times by maximising the cross correlation between the two series.

For each trial, time-matched series of N = 512 consecutive stride times from the left foot were taken from each device. This met guidelines on series length for the calculation of DFA-α [32]. To ensure a series length of N = 512 for all devices, additional unmatched stride times were appended to RunScribe™ series to compensate for missing data.

For each series, the mean, CV and DFA-α were calculated. DFA-α was found using the evenly spaced averaged algorithm [22], with window sizes ranging from 4 to floor(N/5) and detrending performed using a first-order polynomial. Parameters were selected by implementing the method of Phinyomark et al. (2020) [35] for the evenly spaced averaged algorithm.

### 2.6. Statistical Analysis

Statistical analyses were performed in R statistical computing language (version 4.5.1) using the ‘SimplyAgree’ package (version 0.3.0) [36]. Out of a possible 434 stride-time series (31 participants, 2 days, 7 devices (including 4 Blue Trident sampling rates), 379 were calculated from collected data. Non-calculated stride-time series were due to injury between the days (2 participants), a failure to maintain a running gait with a flight time throughout the recording (5 participant × days), and data recording errors with the Loadapp (4 participant × days) and RunScribe™ (2 participant × days) applications.

The agreement of stride times and stride-time variability measures from each wearable device with the instrumented treadmill was assessed using repeated measures Bland Altman plots with 95% limits of agreement (LOA). Concordance correlation coefficients (*r_c_*) were also calculated for stride-time variability measures. Between-day reliability was calculated for stride-time mean, CV and DFA-α for each device using intraclass correlation coefficients (ICCs) based on single-measure, absolute agreement, 2-way mixed-effects model. *r_c_* and ICC values were classified as poor (<0.5), moderate (0.5–0.75), good (0.75–0.90) or excellent (>0.9) [37]. Standard error of measurement (SEM) and absolute minimum detectable change (MDC) were calculated using the method of Weir (2005) [38].

## 3. Results

### 3.1. Stride-Time Agreement

Stride times from wearable devices demonstrated negligible systematic bias relative to the instrumented treadmill (Table 1). The 95% LOAs were similar for the Loadsol^®^ insoles and Blue Trident IMUs across all sampling frequencies, but greater for the RunScribe™ IMU.

### 3.2. Stride-Time Variability Agreement

**Figure 1:**
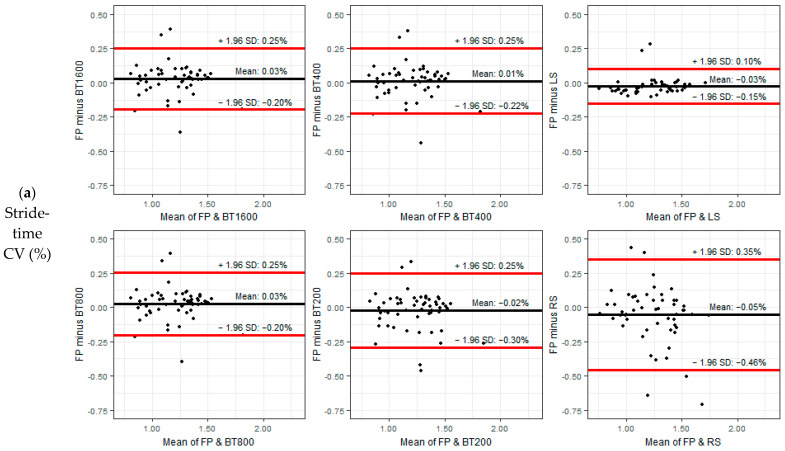

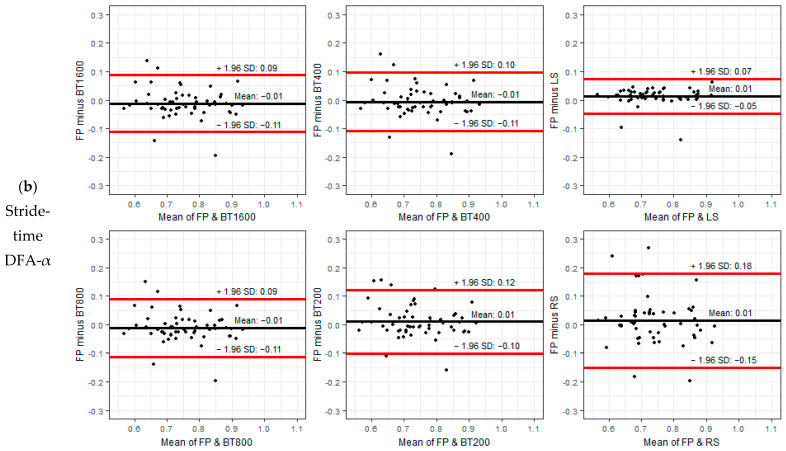
Bland Altman and 95% limits of agreement for (**a**) stride-time coefficient of variation (CV) and (**b**) stride-time detrended fluctuation analysis alpha exponent (DFA-α). Force Plates (FPs); Blue Trident IMU at 1600 Hz (BT1600); Blue Trident IMU at 800 Hz (BT800); Blue Trident IMU at 400 Hz (BT400); Blue Trident at IMU 200 Hz (BT200); Loadsol^®^ insole (LS); RunScribe™ IMU (RS).

All wearable devices displayed a systematic bias in stride-time CV, with both overestimates (Blue Trident at IMU 200 Hz, Loadsol^®^ insoles, RunScribe™ IMU) and underestimates (Blue Trident IMU at 1600 Hz, Blue Trident IMU at 800 Hz, Blue Trident IMU at 400 Hz) observed (Figure 1). For stride-time DFA-α, systematic biases of magnitude 0.01 were observed for all wearable devices, although both overestimates (Blue Trident IMU at 1600 Hz, Blue Trident IMU at 800 Hz, Blue Trident IMU at 400 Hz) and underestimates (Blue Trident at IMU 200 Hz, Loadsol^®^ insoles, RunScribe™ IMU) were observed. For both stride-time CV and DFA-α, the 95% LOAs were smallest for Loadsol^®^ insoles and greatest for the RunScribe™ IMU.

Concordance correlation coefficients for stride-time CV and DFA-α are shown in Table 2. For both measures of variability, Loadsol^®^ insoles demonstrated excellent agreement with the instrumented treadmill, Blue Trident IMUs demonstrated good agreement with the instrumented treadmill across all sampling frequencies, and RunScribe™ IMUs demonstrated moderate agreement with the instrumented treadmill.

### 3.3. Between-Day Reliability

Stride-time mean, CV and DFA-α for each device and on each day are displayed in Table 3. Stride-time mean displayed excellent relative reliability for all devices (ICC ≥ 0.965), whereas stride-time CV displayed moderate-to-good relative reliability (ICC: 0.548–0.808) and stride-time DFA-α displayed poor-to-moderate relative reliability (ICC: 0.469–0.530) with wide ranging confidence intervals (CIs).

## 4. Discussion

This study investigated two issues related to running stride-time analyses using wearable devices by assessing (1) the agreement of stride times and stride-time variability measures between a set of wearable devices and an instrumented treadmill and (2) the between-day reliability of stride-time variability measures. Stride times from all wearable devices displayed negligible systematic bias relative to the instrumented treadmill. However, the agreement of stride-time CV and DFA-α to the instrumented treadmill differed between devices, with the Loadsol^®^ insoles and RunScribe™ IMU displaying the highest and lowest agreement, respectively. Across all devices, stride-time mean, CV and DFA-α displayed excellent, moderate-to-good, and poor-to-moderate relative reliability, respectively.

The stride-time mean, CV and DFA-α in this study were consistent with values previously reported for treadmill running [28,30] and overground running with wearable devices [24,39]. Stride times displayed negligible systematic bias, although the agreement of stride-time CV and DFA-α to the instrumented treadmill varied between devices, with *r_c_* values typically indicating higher agreement of stride-time CV than stride-time DFA-α. Unlike the CV, DFA-α explores the temporal structure of stride-time series and may thus be more sensitive to small temporal differences in event detection timings between measurement devices. Despite that, the mean biases found in stride-time DFA-α were small (|0.01|) and occurred in both directions, suggesting that foot contact timings from wearable devices were not overly contaminated with measurement error noise [23].

Across both variability measures, the Loadsol^®^ insoles had the highest agreement with the instrumented treadmill. In contrast to the stride times calculated from the two IMUs, stride times from the Loadsol^®^ insoles and instrumented treadmill were both calculated using a threshold for vertical force, which may explain the higher agreement observed with this device. Conversely, the RunScribe™ IMU, which provides pre-processed stride-time data, displayed the lowest agreement. Although our method of concatenation of stride-time series from the RunScribe™ IMU was likely robust given that our stride-time series displayed statistical persistence [34], the artificial jumps it created may have biased our results, particularly for DFA-α which measures long-range correlation properties. Nevertheless, given that differences in DFA-α of magnitude 0.10–0.17 have distinguished between histories of previous injury [15] and training states [40], the 95% LOAs for the RunScribe™ IMU found in the current study indicate that this device, with its tendency to skip strides, is inappropriate for such analyses.

Across sampling frequencies, the agreement between the Blue Trident IMU and the instrumented treadmill was consistent, except for the lowest sampling frequency (200 Hz) analysed. Sampling frequency determines the temporal resolution of stride-time series and should be set relative to the expected variance in stride times [23]. Previous research using force-sensitive resistors also found a minimum sampling frequency of 400 Hz ensured no within-device discrepancies in running stride-time DFA-α [24], which is higher than the minimum sampling frequency of 120 Hz suggested for walking analyses [23]. We found that a sampling frequency of 200 Hz occasionally failed to capture the same peak in resultant acceleration as higher sampling frequencies. This led to changes in sign, but not magnitude, of the mean bias for stride-time CV and DFA-α, larger 95% LOAs for stride-time CV, and lower r_c_ values for both stride-time CV and DFA-α. Although the Loadsol^®^ insoles captured at 200 Hz and displayed higher agreement than the Blue Trident across all sampling frequencies, this may have been even higher if 200 Hz was not the maximum device sampling rate.

Between-day reliability of stride-time mean was excellent (ICC ≥ 0.965). Furthermore, between-day reliability of stride-time CV ranged from moderate to good (ICC: 0.548–0.808) and between-day reliability of stride-time DFA-α ranged from poor to moderate (ICC: 0.469–0.530). We previously suggested that different stride-time measurement methodologies may be the cause of the reported differences in the between-day reliability of stride-time variability measures [30]. ICC point estimates for stride-time CV have been reported to range from poor to good when using RunScribe™ IMUs [30], as moderate when using an Optogait system [28], to range from moderate to good when using force-sensitive resistors [29], and as good when using motion-capture-based methods [31]. ICC point estimates for stride-time DFA-α have been reported as poor when using RunScribe™ IMUs [30], as good when using an Optogait system [28] and force-sensitive resistors [29], and to range from poor to good as running duration increases when using motion-capture-based methods [31]. However, the narrower range of variability values found across a suite of measurement devices in the concurrent data collection in our study does not support our previous explanation that differences in between-day reliability are device-related. These data suggest that between-day differences seen in this, and previous studies, largely reflect natural day-to-day biological variation and that measurement errors have a smaller influence. This may be particularly important for stride-time DFA-α, where the lower between-day reliability suggests that true longitudinal changes over time may be more difficult to detect given the magnitude of day-to-day biological variation. Nevertheless, given the previous success of stride-time variability measures in detecting fatigue during a prolonged run [39], cumulative fatigue during overload training [40] and previous injury [15], they remain a valuable tool, although the rolling mean of stride-time variability values may be required to monitor changes over time [24].

This study had several limitations. Firstly, conditions were limited to fixed speed treadmill running so that the agreement of wearable devices against the instrumented treadmill could be assessed. Thus, our interpretations may not apply to other running environments or other speeds. Secondly, participants were likely unfamiliar with running on a tandem instrumented treadmill prior to the study. Although they completed a period of familiarisation in line with guidelines for novice treadmill runners [19], there may have been a small learning effect from the first to the second day.

## 5. Conclusions

This study investigated the agreement and between-day reliability of stride-time variability measures from a set of wearable devices. Loadsol^®^ insoles, Blue Trident IMUs and RunScribe™ IMUs display excellent, good and moderate agreement with an instrumented treadmill, respectively, for stride-time CV and DFA-α, highlighting the suitability of selected wearable devices for measuring running gait variability in overground running conditions. However, stride-time CV and DFA-α displayed moderate-to-good and poor-to-moderate relative reliability respectively, indicating the need for caution when monitoring these measures over time. Future research should explore the effects of environmental constraints on stride-time variability by utilising wearable devices in representative overground environments.

## Figures and Tables

**Table 1:** Bland Altman bias and 95% limits of agreement (LOA) for stride times from each wearable device relative to the instrumented treadmill.

	BT1600	BT800	BT400	BT200	LS	RS
Bias (ms)	0.004	0.004	0.005	0.004	−0.171	0.028
95% LOA (ms)	−6.021 to 6.029	−6.115 to 6.124	−6.499 to 6.510	−7.745 to 7.754	−6.186 to 5.845	−21.858 to 21.914

Blue Trident IMU at 1600 Hz (BT1600), Blue Trident IMU at 800 Hz (BT800), Blue Trident IMU at 400 Hz (BT400), Blue Trident IMU at 200 Hz (BT200), Loadsol^®^ insoles (LS), RunScribe™ IMU (RS).

**Table 2:** Concordance correlation coefficients, *r_c_*, and 95% confidence limits for comparison of stride-time variability measures from each wearable device to the instrumented treadmill.

	BT1600 (95% CI)	BT800 (95% CI)	BT400 (95% CI)	BT200 (95% CI)	LS (95% CI)	RS (95% CI)
CV	0.859 (0.790, 0.907)	0.856 (0.784, 0.905)	0.848 (0.773, 0.900)	0.807 (0.722, 0.868)	0.951 (0.920, 0.970)	0.642 (0.527, 0.734)
DFA-α	0.848 (0.784, 0.894)	0.847 (0.782, 0.893)	0.846 (0.783, 0.892)	0.820 (0.754, 0.869)	0.935 (0.899, 0.958)	0.623 (0.499, 0.721)

CV: Coefficient of Variation; DFA-α: Detrended Fluctuation Analysis alpha exponent; Blue Trident IMU at 1600 Hz (BT1600); Blue Trident IMU at 800 Hz (BT800); Blue Trident IMU at 400 Hz (BT400); Blue Trident at IMU 200 Hz (BT200); Loadsol^®^ insole (LS); RunScribe™ IMU (RS).

**Table 3:** Between-day reliability of stride-time mean, CV and DFA-α.

		Day 1 Mean (95% CI)	Day 2 Mean (95% CI)	ICC (95% CI)	SEM	MDC
Mean (ms)	FP	728.0 (710.5, 745.4)	726.2 (708.2, 744.2)	0.970 (0.943, 0.984)	8.1	21.1
	BT1600	728.0 (710.5, 745.4)	726.2 (708.2, 744.2)	0.970 (0.943, 0.984)	8.1	21.1
	BT800	728.0 (710.5, 745.4)	726.2 (708.2, 744.2)	0.970 (0.943, 0.984)	8.1	21.1
	BT400	728.0 (710.5, 745.4)	726.2 (708.2, 744.2)	0.970 (0.943, 0.984)	8.1	21.1
	BT200	728.0 (710.5, 745.4)	726.2 (708.2, 744.2)	0.970 (0.943, 0.984)	8.1	21.1
	LS	728.4 (709.5, 747.4)	726.8 (707.2, 746.4)	0.970 (0.941, 0.985)	8.1	22.1
	RS	724.9 (707.9, 741.9)	723.0 (705.4, 740.7)	0.965 (0.933, 0.982)	8.1	22.1
CV (%)	FP	1.204 (1.126, 1.283)	1.219 (1.115, 1.324)	0.695 (0.478, 0.832)	0.131	0.351
	BT1600	1.173 (1.098, 1.247)	1.206 (1.097, 1.316)	0.555 (0.283, 0.745)	0.161	0.431
	BT800	1.175 (1.101, 1.249)	1.209 (1.099, 1.320)	0.548 (0.275, 0.740)	0.161	0.441
	BT400	1.186 (1.112, 1.260)	1.223 (1.113, 1.334)	0.567 (0.301, 0.752)	0.151	0.431
	BT200	1.230 (1.148, 1.312)	1.257 (1.145, 1.369)	0.665 (0.436, 0.813)	0.141	0.391
	LS	1.232 (1.152, 1.312)	1.244 (1.135, 1.353)	0.682 (0.446, 0.829)	0.131	0.361
	RS	1.259 (1.155, 1.364)	1.286 (1.154, 1.418)	0.808 (0.653, 0.898)	0.131	0.351
DFA-α	FP	0.729 (0.693, 0.765)	0.768 (0.732, 0.805)	0.476 (0.191, 0.690)	0.061	0.171
	BT1600	0.743 (0.708, 0.779)	0.775 (0.731, 0.819)	0.495 (0.216, 0.702)	0.071	0.191
	BT800	0.743 (0.707, 0.778)	0.774 (0.729, 0.818)	0.498 (0.220, 0.705)	0.071	0.191
	BT400	0.737 (0.702, 0.773)	0.767 (0.723, 0.812)	0.503 (0.225, 0.708)	0.071	0.191
	BT200	0.720 (0.681, 0.758)	0.752 (0.709, 0.795)	0.530 (0.260, 0.726)	0.071	0.191
	LS	0.716 (0.678, 0.754)	0.754 (0.716, 0.792)	0.469 (0.172, 0.692)	0.061	0.181
	RS	0.707 (0.666, 0.747)	0.757 (0.711, 0.802)	0.480 (0.184, 0.697)	0.071	0.201

CV: Coefficient of Variation; DFA-α: Detrended Fluctuation Analysis alpha exponent; FP: Force plates in instrument treadmill; Blue Trident IMU at 1600 Hz (BT1600); Blue Trident IMU at 800 Hz (BT800); Blue Trident IMU at 400 Hz (BT400); Blue Trident at IMU 200 Hz (BT200); Loadsol^®^ insoles (LS); RunScribe™ IMU (RS); ICC: Intraclass correlation coefficient; SEM: Standard Error of Measurement; MDC: Minimum Detectable Change.

## Data Availability

Data is contained within the article.
